# Transnasal PLGA Nanoparticles with Terpene Permeation Enhancers: Membrane Remodeling and Tight Junction Modulation for Enhanced Brain Drug Delivery

**DOI:** 10.3390/ijms26083861

**Published:** 2025-04-18

**Authors:** Yi Zhang, Zishuo Guo, Haitong Zhang, Hongmei Wei, Tieshan Wang, Shouying Du, Pengyue Li

**Affiliations:** School of Chinese Materia Medica, Beijing University of Chinese Medicine, Beijing 102488, China; zhangyi170605@126.com (Y.Z.); 20240941524@bucm.edu.cn (Z.G.); 20230935108@bucm.edu.cn (H.Z.); 20240935123@bucm.edu.cn (H.W.); 700612@bucm.edu.cn (T.W.)

**Keywords:** PLGA nanoparticles, blood–brain barrier, curcumol, menthol, intranasal administration

## Abstract

Nasal nanodrug delivery has gained prominence as a non-invasive method for administering therapeutic agents to the brain. However, the limited nasal cavity volume and the low drug loading capacity of nanoparticles contribute to a reduced accumulation of the drug within the brain tissue. Therefore, the aim of the present study was to investigate the role of the drug delivery combination “transnasal route + nanoparticle drug delivery system + chemical osmosis technology” in promoting drug accumulation in the brain. We constructed an in vitro olfactory sheath cell model based on the direct nose–brain pathway and a vascular endothelial cell model based on the indirect pathway, and investigated the transport behaviors and mechanisms of Poly(lactic-co-glycolicacid)-Nanoparticles (PLGA-NPs) in combination with two terpene aroma constituents (menthol and curcumol). Menthol and curcumol significantly improved the intracellular accumulation of PLGA-NPs, which may be related to changes in the endocytosis pathway and intercellular tight junction proteins. Meanwhile, the results of laser scanning confocal microscopy and atomic force microscopy showed that menthol and curcumol disrupted different tight junction proteins of vascular endothelial cells, and the biomechanical properties (e.g., rigidity and roughness) of the olfactory sheath cells and vascular endothelial cell cytomembranes were also greatly changed. The delivery system of “transnasal route + nanoparticle drug delivery system + chemical osmosis technology” has great potential for intranasal delivery of drugs for the treatment of brain diseases.

## 1. Introduction

The World Health Organization’s 2021 Statistical Yearbook shows that brain diseases, such as strokes, are the second leading cause of death worldwide [1]. The number of patients with these diseases is expected to increase significantly as the population ages and existing treatments have significant limitations [2,3,4]. Of the 37 new therapies approved by the FDA in 2022, almost all treatments only temporarily delay the onset of neurological problems [5]. At the same time, the blood–brain barrier (BBB) makes it difficult to deliver drugs [6,7]. Almost all large molecules and most water-soluble molecules are unable to enter the central nervous system (CNS) after systemic administration [8]. In this context, nasal drug delivery has become an important research direction for brain-targeted therapies because of its noninvasive nature and high safety [9,10]. It is now widely accepted that there are two main pathways for substances entering the brain through the nasal cavity: (a) direct nasal–brain pathway through the olfactory/trigeminal nerves [11]; (b) indirect nasal–brain pathway via BBB permeation [12]. However, nasal drug delivery systems face many challenges. The nasal cavity has a small volume, and it can only hold 25–200 μL of liquid or 25 milligrams of powder [13]. In addition, the cilia of the nasal mucous membrane rapidly clear the fluid. This means that drugs that are not absorbed in time are easily transferred to the nasopharynx [14]. Studies have confirmed that particle size control is a key parameter in optimizing the efficiency of nasal drug delivery. The construction of a nano-delivery system not only reduces the size of the drug to prolong its retention in the mucous membranes, but also enhances the stability of the drug and ultimately improves the concentration of the drug in the target area of the brain [15].

PLGA is a type of biodegradable polymer approved by the FDA. It is made by combining lactic acid and glycolic acid. This material is very compatible with living tissue and breaks down easily, which makes it useful in medical applications, especially in targeted drug delivery systems [16,17]. PLGA nanoparticles have been widely studied as a way to deliver drugs through the nose. PLGA-NPs have a large specific surface area and surface modification of NPs by targeting ligands and polymers, which improves drug delivery, cellular uptake, and bioavailability at the target site [18]. PLGA-NPs achieve brain-targeted delivery of therapeutic drugs for neurological diseases through the intranasal route of administration, and show unique therapeutic advantages in the intervention of central nervous system diseases such as Alzheimer’s disease, Parkinson’s disease, brain tumors, cerebral ischemia, epilepsy, depression, and schizophrenia [19]. Tretinoin (extracted from Radix Rehmanniae) was delivered to the brain via intranasal (IN) administration of Tween-80, CS hydrochloride, ice/peppermint eutectic mixture, and PEG-modified multi-coated PLGA nanoparticles (NPs) for the treatment of Alzheimer’s disease, and in vitro experiments have shown that it possesses good transmembrane permeability and slow-release properties to Caco-2 cells [20]. The research team’s objective is to develop lactoferrin-loaded rotavirus-PLGA nanoparticles for targeted drug delivery in the striatum of a Parkinson’s disease model [21]. Through intranasal administration, arginyl-glycyl-aspartic tripeptide (RGD) conjugated paclitaxel (PTX)-loaded nanoparticles significantly reduce tumor burden by triggering apoptosis and suppressing cancer cell proliferation, while preserving the G0 phase in normal brain cells [22].

Although nanodelivery systems have shown potential in the treatment of brain diseases, their inherent low drug loading capacity often makes it difficult to achieve the desired efficacy [23]. To overcome this limitation, researchers have turned their attention to BBB modulation strategies reversing the opening of the BBB by increasing the intracerebral delivery efficiency of nanoparticles [24]. Chinese herbal aromatic permeation enhancers (also known as messenger drugs) have attracted attention for their unique advantages. Studies have shown that these ingredients are not only safe as brain permeation enhancers (BPEs), but also guide drugs to the brain with good brain-targeting properties [25]. The current mainstream method of constructing the coupling system is by physically mixing BPEs with drug delivery systems [26].

Menthol, a primary component of peppermint oil, is a cyclic monoterpene known for its unique pharmacological activity [27]. The significant advantage of menthol’s application as an osmotic stimulant is its excellent safety profile, with studies showing menthol’s neuroprotective effects [28]. Research data show that the substance has a very weak acute toxicological response and does not present a carcinogenicity risk [29]. In terms of genotoxicity assessment, menthol did not show genetic material damage in a number of standardized tests, including the Ames test and chromosomal abnormality detection using Chinese hamster ovary cells [30]. Preliminary studies have shown that menthol enhances the permeability of geraniol in nasal administration and facilitates the transport of geraniol from the nose to the brain [31]. Menthol-bovine serum albumin modified silver nanoparticles (MBS-Abz) developed by Liang’s team confirmed that menthol significantly enhances the BBB penetration efficiency of albendazole [32]. Reverse micelle (RM)-encapsulated veratridin lipid nanocapsules (LNC) nanocomplexes combined with menthol significantly enhance BBB penetration efficiency and neuroprotective activity, providing a novel drug delivery strategy for Alzheimer’s disease treatment [33]. Multiple mechanisms of action revealed by in-depth studies included prolongation of nasal retention time, down-regulation of tight junction protein expression, and expansion of the cell gap synergistically promote nose-to-brain transport [34]. Curcumol is also a sesquiterpenoid compound found in oils of curcuma species, like curcuma kwangsiensis by S.G.Lee and C.F.Liang, and curcuma wenyujin by Y.H.Chen et C. Ling. Curcumol has anticancer, antibacterial, antifungal, antiviral, and anti-inflammatory physiological activities [35]. Repeated toxicity studies in female and male rats orally administered different doses of curcumol showed that no clinical signs of toxicity or changes in individual body weight and food consumption were observed [36]. Currently, studies on curcumol have focused on its anticancer activity, and fewer studies have been conducted on its nasal permeability promotion [37].

The aim of this experiment was to investigate whether natural terpenoids such as menthol and curcumol can enhance the transport of PLGA-NPs to the brain after intranasal administration. The following objectives were hoped to be achieved through the following study: (a) whether natural permeation enhancers such as menthol/curcumol can promote the transport of PLGA-NPs to the brain; and (b) to explore the underlying mechanisms of enhanced brain transport.

## 2. Results and Discussion

### 2.1. Characterization of PLGA-NPs

In this study, PLGA-NPs prepared via the emulsion–solvent evaporation method exhibited a yellowish-green color attributed to FITC. The nanoparticles demonstrated excellent dispersion, with no signs of delamination or precipitation observed.

**Figure 1:**
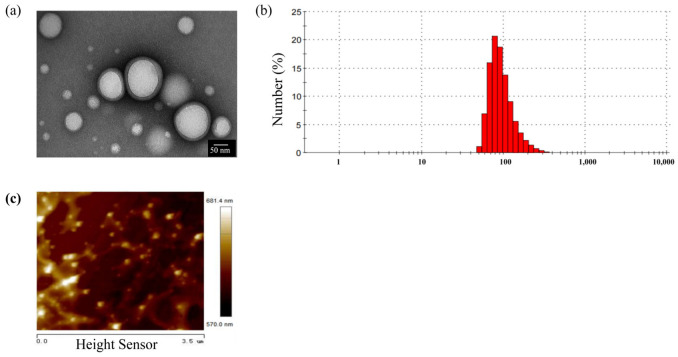
Physical characteristics of PLGA-NPs. (**a**) TEM images (Scale bar: 50 nm); (**b**) particle size distribution of PLGA-NPs; (**c**) the rigidity of PLGA-NPs detected by AFM (Scale bar: 1 μm).

As shown in Figure 1a, PLGA-NPs displayed a spherical morphology with smooth surfaces and uniform dispersion. TEM revealed a particle size of approximately 100 nm, consistent with Malvern laser particle size analysis results (98.7 ± 3.6 nm) (Figure 1b). The polydispersity index (PDI) was 0.1 ± 0.0, indicating homogeneous size distribution without aggregation. The zeta potential measured −4.0 ± 1.3 mV, confirming slight negative surface charge.

Atomic force microscopy (AFM) analysis (Figure 1c) showed uniformly distributed spherical PLGA-NPs with an average Young’s modulus of 104.5 ± 8.8 MPa, directly quantifying nanoparticle stiffness.

### 2.2. Biocompatibility Studies of PLGA-NPs

**Figure 2:**
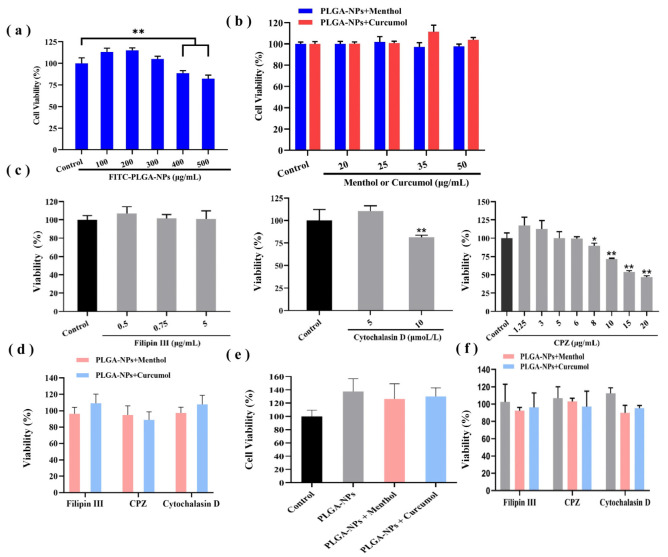
Cytotoxicity study of bEnd.3 cells and OECs. (**a**) Effects of PLGA-NPs with different concentrations on the bEnd.3 cells viability for 12 h (*n* = 5). (**b**) Effects of PLGA-NPs with different concentrations of menthol or curcumol on the bEnd.3 cells viability for 12 h (*n* = 5). (**c**) Effects of endocytosis inhibitors with different concentrations on the bEnd.3 cells viability for 8 h (*n* = 5). (**d**) Effects of endocytosis inhibitors combined with PLGA-NPs + menthol or curcumol on the bEnd.3 cells viability for 8 h (*n* = 5). (**e**) Effects of PLGA-NPs with menthol or curcumol on the viability of OECs (*n* = 3). (**f**) Effect of endocytosis inhibitors combined with PLGA-NPs + menthol or curcumol on the viability of OECs (*n* = 3). * *p <* 0.05 vs. Control group, ** *p <* 0.01 vs. Control group.

The cytotoxicity of PLGA-NPs was evaluated on bEnd.3 cells and OECs using the MTT assay. The results showed a correlation between the effect of PLGA-NPs on cell viability in both bEnd.3 cells and OECs and their concentration. The damage to cell viability was found to increase with the rising concentration of the administered substance when the concentrations exceeded 300 μg/mL. Therefore, the concentration of PLGA-NPs was determined within a range of 100 to 300 μg/mL. Considering both the preparation and dosage requirements, a final concentration of 100 μg/mL was selected for subsequent experiments. The details are shown in Figure 2a in the text.

The cytotoxicity results of PLGA-NPs in conjunction with aromatic components are presented in Figure 2b. The safe concentration of menthol was determined to be 50 μg/mL when combined with PLGA-NPs (100 μg/mL) (Figure 2b). The curcumol group (50 μg/mL) represented excellent biocompatibility with a higher cell survival rate. As illustrated in Figure 2c, the concentrations of each inhibitor were selected as follows: Filipin Ⅲ (5 μg/mL), Cytochalasin D (5 μmol/L), and CPZ (6 μg/mL). The results presented in Figure 2d indicate that the survival rates of bEnd.3 cells across all groups surpassed 85%, thereby fulfilling the experimental requirements.

The formulation concentrations mentioned above were also applicable to OECs, as shown in Figure 2e,f.

### 2.3. Study on the Transport Behavior of PLGA-NPs Combined with Aromatic Components

#### 2.3.1. PLGA-NPs Transport Across the BBB Monolayer

**Figure 3:**
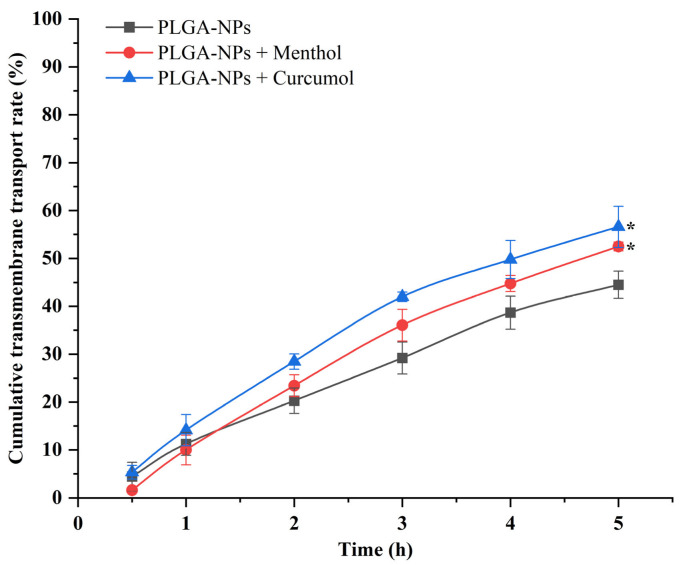
Effect of aromatic components on the transport rate of PLGA-NPs across the cell monolayer of bEnd.3 cells. * *p <* 0.05 compared with PLGA-NPs Group.

The Papp value obtained from the transport experiment of PLGA-NPs combined with menthol or curcumol were 14.0651 × 10^−6^ cm/s and 14.2412 × 10^−6^ cm/s, respectively. These values were significantly higher than that of PLGA-NPs group (11.2621 × 10^−6^ cm/s), which confirmed that both of them could promote the transmembrane transport of PLGA-NPs and have a certain enhancement effect on their permeability as shown in Figure 3.

#### 2.3.2. The Cellular Uptake Behavior of PLGA-NPs in bEnd.3 Cells and OECs

**Figure 4:**
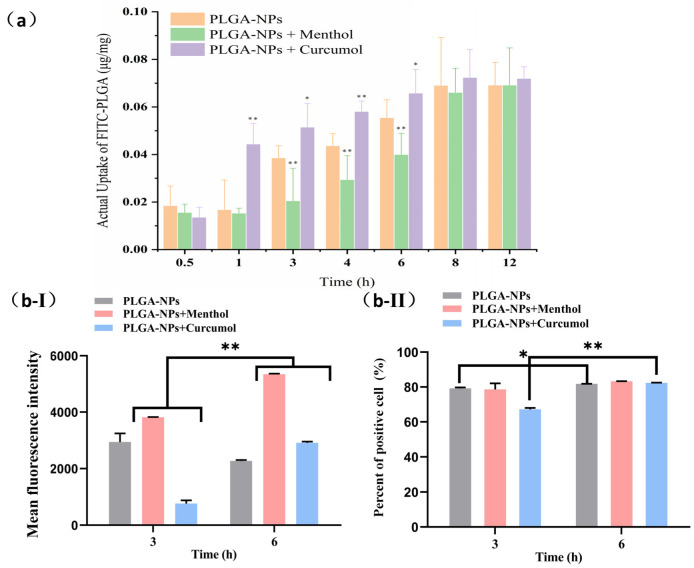
The cellular uptake behavior of PLGA-NP by bEnd.3 cells and OECs. (**a**) The effect of menthol and curcumol on the uptake of PLGA-NPs by bEnd.3 cells (*n* = 3). (* *p* < 0.05, ** *p* < 0.01 compared with PLGA-NPs group at the same time point). (**b**) Effect of menthol and curcumol on the uptake of PLGA-NPs by OECs (*n* = 4). (* *p* < 0.05, ** *p* < 0.01 vs. the group at 3 h, in which (**b-I**) was the MFI diagram, and (**b-II**) was the percentage of positive cells).

We carried out experiments to investigate the effects of compatible aromatic components on the uptake of PLGA-NPs by bEnd.3 cells. As shown in Figure 4a, the uptake of PLGA-NPs by bEnd.3 cells in the PLGA-NPs group, PLGA-NPs + Curcumol group and PLGA-NPs + Menthol group, initially increased and then tended to balance over time. A state of equilibrium was reached from 8 to 12 h, no significant differences in uptake were observed among the three groups (*p >* 0.05). From 1 to 6 h, the uptake of PLGA-NPs was ranked as follows: PLGA-NPs + Curcumol group> PLGA-NPs group > PLGA-NPs + Menthol group.

The same experiments were performed with OECs. Due to the high cost of animal extraction, primary cells from OECs were studied at two time points (3 h and 6 h). As shown in Figure 4b-Ⅰ, there was no significant difference (*p* > 0.05) in the mean fluorescence intensity (MFI) of the PLGA-NPs group at 6 h compared to the 3 h time point. Upon binding PLGA-NPs to aromatic components, MFI increased over time (*p* < 0.01). Notably, the MFI of the cells to PLGA-NPs was higher than that of the PLGA-NPs alone group after 6 h. After the treatment lasted for 3 h, menthol was found to enhance the uptake of PLGA-NPs by the cells, while at 3 h, the MFI of the PLGA-NPs + curcumol group was lower than that of the PLGA-NPAs group. As shown in Figure 4b-Ⅱ, the percentage of positive cells in the PLGA-NPs + curcumol group was lower than that in the PLGA-NPs group at 3 h, whereas no difference was observed at 6 h.

### 2.4. Mechanism Study of PLGA-NPs Transport Combined with Aromatic Components

#### 2.4.1. Expression of the TJ-Associated Proteins

**Figure 5:**
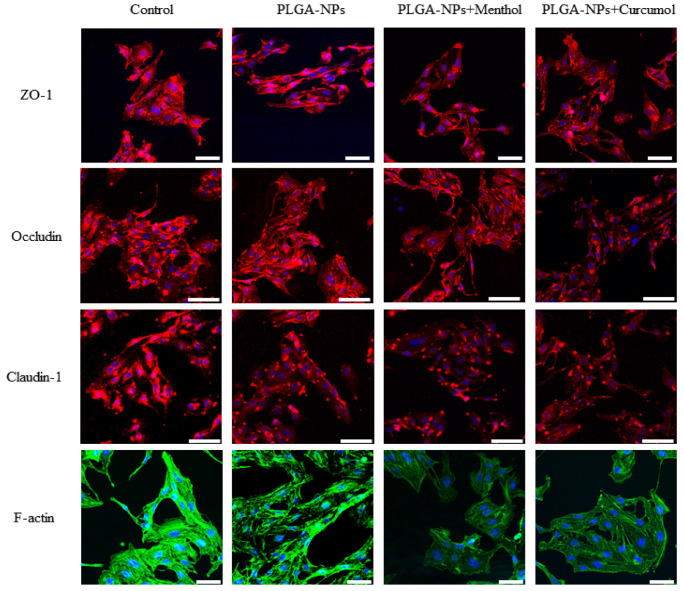
Effect of PLGA-NPs combined with menthol or curcumol on the tight junction proteins in bEnd.3 cells monolayer (the scale bars of ZO-1 and F-actin correspond to 50 μm; the scale bars of Occludin and Claudin-1 correspond to 75 μm).

The effects of PLGA-NPs before and after the dosing of menthol or curcumol on the four tight junction proteins, ZO-1, Occludin, Claudin-1, and F-actin, in bEnd.3 cells are shown in Figure 5 and Table 1.

The fluorescence images of the tight junction proteins are shown in Figure 5. All four proteins were either absent in certain cells or showed fragmented and plaque-like accumulation, to some extent, when PLGA-NPs were combined with menthol or curcumol. The fluorescence intensity of both Claudin-1 and F-actin decreased when bEnd.3 cells monolayers were co-incubated with PLGA-NPs. A more pronounced reduction in fluorescence intensity was observed for all four proteins when PLGA-NPs were combined with menthol or curcumol.

As illustrated in Table 1, PLGA-NPs had no significant effect on the expression of the tight junction proteins ZO-1 and Occludin. However, they exhibited a notable inhibitory effect on the expression of Claudin-1 and F-actin in bEnd.3 cells (*p* < 0.01). When combined with the directional ingredient menthol, PLGA-NPs demonstrated a certain degree of inhibition on all four proteins. Additionally, when paired with curcumol, there was a significant inhibitory effect observed on ZO-1, Occludin, and Claudin-1.

#### 2.4.2. Studies on Interventions for Endocytic Pathways

**Figure 6:**
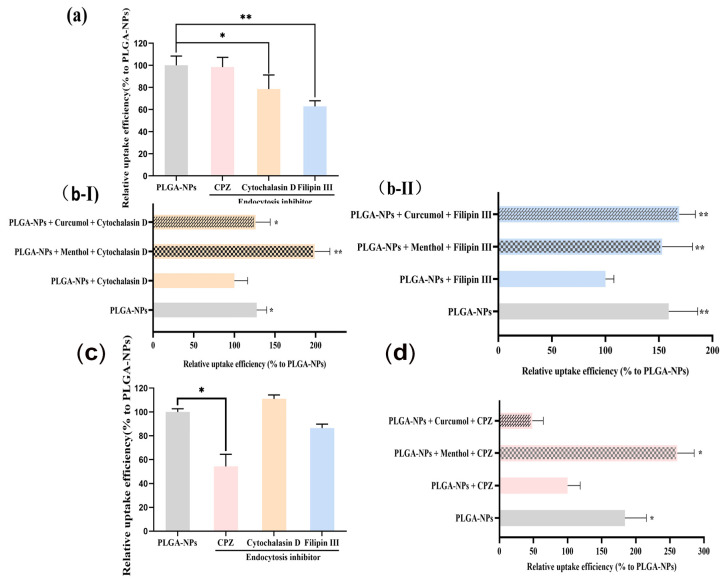
Effects of aromatic components on endocytic pathways of bEnd.3 cells and OECs. (**a**) Effects of endocytosis inhibitors on cellular uptake of PLGA-NPs by bEnd.3 cells (*n* = 3). * *p* < 0.05, ** *p* < 0.01 vs. PLGA-NPs Group (without endocytosis inhibitors). (**b**) Effects of aromatic components (menthol and curcumol) on the main pathway of bEnd.3 cells (*n* = 3). (**b-I**) Macropinocytosis. (**b-II**) Caveolae-mediated endocytosis. * *p* < 0.05, ** *p* < 0.01 vs. PLGA-NPs + Cytochalasin D Group or PLGA-NPs + Filipin III Group. (**c**) Effects of endocytosis inhibitors on cellular uptake of PLGA-NPs by OECs (*n* = 4). * *p* < 0.05, ** *p* < 0.01 vs. PLGA-NPs Group that did not contain endocytosis inhibitors. (**d**) Effects of aromatic components (menthol and curcumol) on the main pathway of OECs (*n* = 4). * *p* < 0.05 vs. PLGA-NPs + CPZ Group.

In the indirect nasal–brain pathway model (bEnd.3 cells), Cytochalasin D and Filipin III significantly inhibited PLGA-NP internalization by 21.49% and 37.18%, respectively (Figure 6a). Menthol and curcumol reversed this inhibition, increasing PLGA-NP accumulation (Figure 6b). In the direct pathway model (OECs), chlorpromazine (CPZ) reduced uptake by 45.66% (Figure 6c). Menthol counteracted CPZ-induced inhibition, elevating fluorescence intensity in the PLGA-NPs + Menthol + CPZ group (Figure 6d), while curcumol showed no significant effect.

#### 2.4.3. Biomechanical Properties Assessment of bEnd.3 Cells and OEC

The biomechanical properties of cells play a crucial role in their biological functions. Whether it involves maintaining cell morphology or facilitating physiological processes, these properties are significantly influenced by mechanical signals [38]. The rigidity of cells is primarily determined by the structure of the cytoskeleton [39]. Disruption of the cytoskeleton can not only affect the process of cellular uptake, but also interfere with the tight junctions among cells. Consequently, studying the biomechanical properties of cells is essential for elucidating the transport mechanism of PLGA-NPs when they are associated with aromatic components. In this study, surface morphology, surface roughness, and Young’s modulus of cells were investigated to assess the biomechanical properties of bEnd.3 cells and OEC cells.

**Figure 7:**
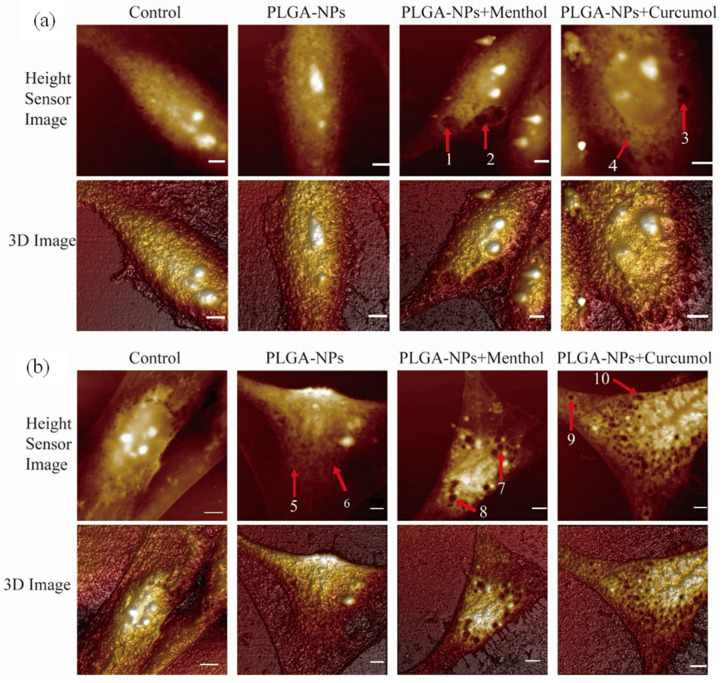
Cytomembrane morphology of bEnd.3 cells and OECs co-incubated with PLGA-NPs and aromatic components (Scale bar: 5 μm). (**a**) Cytomembrane morphology of bEnd.3 cells. (**b**) Cytomembrane morphology of OECs. 1–10 represent the pores caused by PLGA-NPs group, PLGA-NPs+Menthol group, and PLGA-NPs+Curcumol group on the cell membrane surface of bEnd.3 cells and OECs. The red arrows represent the locations of the pores.

Surface morphology analysis via atomic force microscopy (AFM) revealed distinct structural changes in both indirect and direct pathway models (Figure 7). AFM images showed a gradient in height from the nucleus to the cell membrane, with higher brightness corresponding to greater elevation. In the indirect pathway (bEnd.3 cells), the control group exhibited smooth membranes with minimal pores, while PLGA-NP-treated cells displayed densely distributed small pores (depth: 23.7 ± 4.3 nm). Coupling PLGA-NPs with aromatic components further altered morphology: PLGA-NPs + menthol produced larger pores (e.g., pore 1: 294.94 nm, pore 2: 206.10 nm), whereas PLGA-NPs + curcumol increased pore depth (e.g., pore 3: 223.42 nm, pore 4: 63.38 nm).

For the direct pathway (OECs), PLGA-NPs induced deeper pores compared to bEnd.3 cells (pore 5: 78.06 nm, pore 6: 193.73 nm). Menthol-enhanced formulations generated ultradeep pores (pore 7: 376.33 nm, pore 8: 312.74 nm), while curcumol-treated groups showed pores up to 221.13 nm (pore 10). These findings demonstrate that aromatic components significantly expand pore dimensions, suggesting enhanced membrane permeability for PLGA-NP transport.

The surface roughness and Young’s modulus of bEnd.3 cells and OECs are shown in Table 2. Root mean square roughness (Rq) and roughness average (Ra) were employed to characterize the roughness of cell membrane, and Young’s modulus was used to characterize the rigidity of the membrane.

For the model of direct nose-to-brain pathway, the roughness of bEnd.3 cells were significantly increased, and this increase was further enhanced following the administration of PLGA-NPs in combination with menthol or curcumol. Similar results were observed in OECs.

Interestingly, when compared with the control group, Young’s modulus of the PLGA-NPs group exhibited a slight increase. However, upon co-administration of PLGA-NPs with menthol or curcumol, there was a significant reduction in Young’s modulus. This finding indicated that menthol and curcumol may enhance the flexibility of cytomembranes, potentially due to their effects on endocytosis pathways.

## 3. Discussion

The particle size of PLGA-NPs prepared in this study is consistent with the reported optimal particle size range of nanomedicine delivery systems, which can enhance brain drug concentration [40]. This size range facilitates efficient cellular uptake and BBB penetration. Young’s modulus, a critical parameter for nanoparticle mechanical properties, reflects material resistance to deformation under stress. The measured value (104.5 MPa) provides insights into PLGA-NP rigidity, which may influence biodistribution and cellular interactions [41,42]. The slight negative zeta potential (−4.0 mV) suggests minimal electrostatic repulsion, supporting colloidal stability as evidenced by low PDI (0.1). These properties collectively validate the suitability of PLGA-NPs for targeted drug delivery. The oil–water partition coefficient (ALog P) is a significant indicator of molecular hydrophobicity. Calculations based on the Ghose–Crippen method of the Traditional Chinese Medicine Systematic Pharmacology Database and Analytical Platform (TCMSP) demonstrated that menthol (molecular weight 156.27 g/mol, ALog P 2.78) and curcumol alcohols (molecular weight 236.33 g/mol, ALog P 2.79) were found to be lipophilic and aromatic in character, and exhibited good permeability to biofilms [43]. Moreover, several reports have confirmed that menthol could promote the permeability of biological barriers [44,45]. The experimental results indicate that both compounds promote the transmembrane transport of PLGA-NPs, possibly by modulating tight junctions or enhancing paracellular diffusion. This mechanistic synergy highlights their potential as permeation enhancers in nanodrug delivery systems.

In cellular pharmacokinetic studies, the uptake of PLGA-NPs in conjunction with curcumol was significantly increased prior to reaching a state of equilibrium. While the actual uptake of the PLGA-NPs + Menthol group was observed to be lower than that of the PLGA-NPs group during the 3–6 h period, the uptake at 8–12 h suggests this discrepancy may be attributed to menthol’s concentration-dependent enhancement of PLGA-NP efflux rates surpassing uptake rates in short timeframes. In the indirect pathway model of nasoencephalic transport, curcumol promotes PLGA-NP uptake by bEnd.3 cells at 1–6 h. In olfactory ensheathing cells (OECs), menthol’s early-phase uptake promotion (3 h) contrasts with curcumol’s delayed effect (6 h), possibly due to divergent interactions with efflux transporters. Curcumol’s reduced MFI at 3 h but increased cell penetration at 6 h implies a biphasic mechanism: initial efflux dominance followed by enhanced paracellular transport.

Tight junctions, formed by proteins anchored to the cytoskeleton, serve as critical barriers of the paracellular pathway [46]. The observed reduction in fluorescence intensity of Claudin-1 and F-actin, particularly when PLGA-NPs were combined with menthol or curcumol, suggested these components might downregulate TJ-associated proteins to induce BBB opening. Notably, menthol exhibited stronger F-actin downregulation compared to curcumol. Zonula occludens-1 (ZO-1), Occludin, and Claudin-1 collectively maintain junctional integrity [47,48,49,50]. Endocytic pathway studies revealed bEnd.3 cells primarily internalize PLGA-NPs through macropinocytosis and caveolae-mediated endocytosis [51,52]. Both menthol and curcumol reversed inhibitor-induced uptake decreases and significantly increased PLGA-NP accumulation, potentially through modulation of these pathways. In OECs, clathrin-mediated endocytosis dominated PLGA-NP uptake, with menthol likely interfering with this pathway.

Biomechanical assessments showed expanded pore sizes in cells treated with menthol (294.94 nm) and curcumol (223.42 nm), correlating with disrupted cytoskeletal integrity and TJ protein downregulation. Menthol’s greater lipophilicity (ALog P 2.78) may enhance lipid bilayer interactions, consistent with its stronger membrane remodeling effects [44].

### Prospects

The present study of the direct nose–brain pathway is based on primary cultured olfactory nerve sheath cell model. Due to technical limitations, the current mechanistic study is not complete, and subsequent experiments can be carried out to investigate the membrane fluidity and lysosomal co-localization. In addition, based on the literature research and online pharmacology database, the target prediction of the effects of the aromatic components menthol and curcumol on the endocytosis pathway of nanocarriers, and the correlation between Piezo-mediated mechano-ion channels and nanocarrier transport behavior can be verified, so as to further explore the mechanism of drug transport at the gene level. Finally, no drug was encapsulated in this experiment to explore the potential of transnasal drug delivery under the effect of menthol/curcumol to promote permeation for the treatment of a nerve center disease, and further experiments can be carried out by encapsulating different drugs in the future.

## 4. Materials and Methods

### 4.1. Materials

Fluorescein isoAthiocyanate (FITC) is mainly used as a fluorescent dye in fluorescent antibody technology and has a bright yellow-green fluorescence. In this experiment, FITC was combined with PLGA for the subsequent cell fluorescence imaging experiments. PLGA-FITC (molecular weight: 15,000, 75:25) was purchased from Xi’an Ruixi biological Co., Ltd. (Xi’an, China). Polyvinyl Alcohol 1788 (PVA) was purchased from Shanghai McLean Biochemical Technology Co. (Shanghai, China). Dichloromethane and ethyl acetate were obtained from Tianjin Damao Chemical Reagent Factory (Tianjin, China). In addition, 3-(4,5-dimethylthiazole-2)-2,5-diphenyltetrazolium bromide (MTT, mass fraction 98%) was bought from Beijing BioDee Biotechnology Co., Ltd. (Beijing, China). Dimethyl sulfoxide (DMSO) was purchased from Sigma-Aldrich (St. Louis, MO, USA). Filipin III, Chlorpromazine and Cytochalasin D were purchased from APExBIO (Houston, TX, USA). L-Menthol was obtained from Beijing Ouhe Technology Co., Ltd. (Beijing, China). Curcumol was obtained from Shanghai Yuanye Biotechnology Co., Ltd. (Shanghai, China). Four percent paraformaldehyde and Donkey Serum stock solution for blocking were provided from Beijing Solarbio Technology Co., Ltd. (Beijing, China). Electron microscope fixative was purchased from Wuhan Servicebio Co., Ltd. (Wuhan, China).

### 4.2. Methods

#### 4.2.1. Preparation and Characterization of Formulations

The synthesis of PLGA-NPs was carried out through an emulsification–solvent–evaporation approach based on previous methods. In the initial step, 25 mg of PLGA-FITC was dissolved in a mixture of 0.7 g dichloromethane and 0.3 g ethyl acetate to create the organic phase. Concurrently, two distinct aqueous phases were prepared by dissolving polyvinyl alcohol (PVA) in deionized water at concentrations of 2% and 0.5%, respectively. During the emulsification process under ice-cooled conditions, 5 mLof the 2% PVA solution was gradually incorporated into the organic phase mixture. This combination underwent intermittent sonication cycles (2-second pulses alternating with 2-second pauses) for 10 min using a 200 W ultrasonic homogenizer. The emulsion was then further stabilized by adding 5 mL of the 0.5% PVA solution followed by identical sonication parameters. The resulting emulsion underwent continuous magnetic stirring at ambient temperature for 6 h to initiate solvent evaporation, followed by 24-h standing to ensure complete removal of organic solvents. It should be noted that while this procedure successfully yielded stable PLGA-NPs suspensions for immediate experimental use, the investigation did not include long-term stability assessments. Therefore, all nanoparticle formulations were utilized within seven days of preparation to ensure experimental consistency.

The menthol/curcumol and PLGA-NPs were mixed by a physical mixing method [26]. However, PLGA-NPs were not loaded with menthol and curcumol. The specific mixing method was as follows: PLGA-NPs were diluted to 100 μg/mL in basal medium, and menthol (or curcumol) was diluted to 20, 25, 35, and 50 μg/mL with 100 μg/mL of PLGA-NPs to thoroughly mix the two.

Subsequently, transmission electron microscopy (TEM) was used to observe the micromorphological structure of PLGA-NPs. The particle size, zeta potential, and polydispersity index (PDI) of PLGA-NPs were measured by Zetasizer Nano ZS (Malvern Instruments Ltd., Worcestershire, UK). The rigidity of PLGA-NPs was measured by an Atomic Force Microscope (AFM, Bruker Ltd., Los Angeles, CA, USA). The AFM samples were prepared by adding sterile ultrapure water to dilute the PLGA-NPs suspension at a ratio of 1:10, and then put on mica sheets to determine the values of Young’s modulus after natural air-drying. The AFM setup condition parameters were loading probe as SNL-10 (No. 3) and probe radius of curvature as 34.1 nm. In addition, the Peak Force tapping technique was used, and Quantitative Nanomechanics Mode (QNM Mode) was selected for the working mode.

#### 4.2.2. Cell Culture of bEnd.3 Cells and OECs

Mouse microvascular cerebral endothelial cells (bEnd.3 cells) purchased from Procell Life Science & Technology Co., Ltd. (Wuhan, China) were cultured in DMEM (Gibco, Thermo Fisher Scientific Ltd., Waltham, MA, USA) supplemented with 10% (*v*/*v*) FBS and 100 U/mL penicillin–streptomycin solution at 37 ± 0.5 °C in a 5% CO_2_ humidified incubator. For uptake and transport experiments, bEnd.3 cells were seeded in 12-well plates with 4–5 × 10^5^ cells or on PET inserts with 5 × 10^5^ cells. The cells were then incubated for 24 h or 14 days, respectively, with fresh media changed every other day, to allow the cells to reach confluence.

Based on prior experiments [53], a primary neural cell model was established to simulate direct nose-to-brain pathways in 24-hour-old Wistar rats (Beijing Vital River Laboratory Animal Technology Co., Ltd., Beijing, China) utilizing their Olfactory Ensheathing Cells (OECs). OECs cultured in DMEM/F12 supplemented with 15% FBS and 2% triple-antibody were separated from olfactory bulbs under sterile conditions after digestion with 0.125% trypsin, purification through a 100-mesh cell sieve, and centrifugation at 400× *g*. After incubation for 18 h, OECs were collected by centrifugation and were co-incubated with P75 antibody to stain for 1 h. Then, the positive target cells were sorted into cell culture plates coated with poly-L-lysine by flow sorter (Beckman Coulter, Inc., Brea, CA, USA). After 5 days of continuous incubation, a large number of free OECs were observed, and the neurofilaments were elongated and interlaced. Purity identification was conducted to use a portion of sorted cells cultured in DMEM/F12 supplemented with 15% FBS, 2% triple-antibody, 2 μm/L forskolin, and 20 ng/mL BPE for 6 days. After digestion with 0.25% trypsin and stained with p75 antibody at 4 °C, OECs were collected by centrifugation at 400× *g*. At this time, the purity of OECs was up to 99.9% by flow cytometry, which could be used as a purification scheme for OECs.

The bEnd.3 cells were cultured in a 1:3 ratio with OECs taken from the olfactory bulb of neonatal rats. On the fifth day of culture, a substantial number of free OECs could be seen, and the neurofilaments were elongated and interlaced, while the purity was up to 99.9%.

#### 4.2.3. Cytoxicity Study

The cytotoxicity was assessed using MTT assay. Briefly, bEnd.3 cells with a density of 5 × 10^3^ cells/well were seeded into 96-well microtiter plates and were treated with different concentrations of of PLGA-NPs, and PLGA-NPs + menthol/curcumol. The safe dose of endocytosis inhibitors on bEnd.3 cells was tested with different concentrations of endocytosis inhibitors in a cell medium containing PLGA-NPs. The cell viability in PLGA-NPs combined with menthol or curcumol was examined by adding endocytosis inhibitors at safe concentrations to the medium. Then, the biocompatibility was tested and the absorbance value was read at 490 nm wavelength. The cell viability was calculated as Equation (1):

(1) $$Cell\;viability\left(\%\right)=\frac{A_{experimental\;group}}{A_{control\;gruop}}\times 100\%$$

in which *A* is the absorbance value.

#### 4.2.4. Transport Across bEnd.3 Cell Monolayers

In this study, after preliminary experimental investigation, the formulation groups were set as follows: I. 100 μg/mL PLGA-NPs; II. 100 μg/mL PLGA-NPs + 50 μg/mL menthol; III. 100 μg/mL PLGA-NPs + 50 μg/mL curcumol.

The in vitro blood–brain barrier (BBB) model was constructed by seeding bEnd.3 cells into PET inserts (Corning, Tewksbury, MA, USA) at a density of 5 × 10^5^ cells. After 14 days, the TEER of the cell monolayer was between 50 and 70 Ω·cm^2^. This was the same as the TEER measured under the same membrane materials that had been reported before. It was also the same as the TEER of primary brain endothelial cells. This suggests that the monolayer model of bEnd.3 cells met the experimental requirements. Subsequently, the leak test and fluorescein sodium permeability test further confirmed that the established cell monolayer model was tightly connected and had good integrity for further permeability study. Only the cell models with TEER meeting the above requirements were selected for transport study during the experiment. The various samples and Hanks’ Balanced Salt Solution (HBSS) were separately added to the donor chambers and receiver chambers. After 0.5, 1, 2, 3, 4, and 5 h, 300 μL of the sample was removed from each receiver chamber for measurement and immediately replaced with fresh HBSS of the same temperature and volume. The fluorescence intensity of FITC-PLGA was measured by a microplate reader at an excitation wavelength of 490 nm and an emission wavelength of 520 nm. Then, the cumulative penetration-time curve of the formulations was drawn, and the apparent permeability coefficient (Papp) was calculated by Equation (2):

(2) Papp=dQdtAC0

where *dQ*/*dt* is the permeability rate (μg/s), V is the volume of receiver chamber solution, C_0_ is the initial formulations concentration (μg/mL) in the donor chamber, and *A* is the surface area (cm^2^) of the monolayer.

#### 4.2.5. Accumulation of PLGA-NPs with Aromatic Components in bEnd.3 Cells and OECs

The bEnd.3 cells showing logarithmic growth were seeded into 12-well plates at a seeding density of 2.5 × 10^5^ cells/mL and were co-cultured with various experimental groups in the section of Samples. After incubation of 0.5, 1, 2, 3, 4, 6, 8, and 12 h, the uptake was terminated with PBS at 4 °C, and then the cells were harvested. After digestion with trypsin, bEnd.3 cells were centrifuged at 1000 r/min and resuspended in PBS to obtain the cell suspension. Cell lysate was obtained by an ultrasonic cell crushing device. The behavior of cellular uptake was analyzed semi-quantitatively by detecting the fluorescence intensity of cell lysates with a microplate reader (SpectraMax i3x, Molecular Devices, Shanghai, China) with an excitation wavelength of 490 nm and an emission wavelength of 520 nm. Meanwhile, another cell lysate of 10 μL was taken into a 96-well microtiter plate to determine the protein content by BCA (Nanjing KeyGen Biotech. Inc., Jiangsu, China). The intracellular PLGA was represented by means of μg (PLGA)/mg (protein).

For the direct pathway model, because the OECs taken from a newborn Wistar rat were extremely low and could not be subcultured, only two time points (3 and 6 h) were set for the uptake experiment. The OECs were sorted into 96 wells with 8 × 10^4^ cells and were replaced with the formulations containing medium. After 3 and 6 h of incubation, respectively, the OECs were digested with 0.25% trypsin, centrifuged at 250× *g*, and resuspended in PBS. Finally, the cell suspension was collected and the fluorescence intensity was detected and analyzed by flow cytometry.

#### 4.2.6. Immunostaining of bEnd.3 Cells

After treatment with each experimental group in the section of samples, bEnd.3 cells cultured in laser confocal Petri dishes were washed, fixed with 4% paraformaldehyde for 15 min, blocked for 30 min, and subsequently incubated with primary antibody against ZO-1 (1:300), Occludin (1:50), or Claudin 1 (1:50) overnight, respectively. Then bEnd.3 cells were treated with the secondary antibody of CoraLite594-conjugated Donkey Anti-Rabbit IgG (1:100; Proteintech Group, Chicago, IL, USA) and Fluoroshield Mounting Medium with DAPI (Abcam, Cambridge, UK). Here, the determination of F-actin was fixed and blocked according to the same method as above. Then, the bEnd.3 cells were permeated with 0.2% Triton X-100 at room temperature and the coralite^®^ 488-labeled phalloidin(Proteintech Group, Chicago, IL, USA) was diluted at 1:100 for incubation with bEnd.3 cells at room temperature before DAPI staining. Immunostaining was detected by a confocal microscope (SP8, Leica, Berlin, Germany) and analyzed with Image J. The wavelengths of excitation were 488 nm and 561 nm.

#### 4.2.7. Endocytic Mechanism of PLGA-NPs with Aromatic Components in bEnd.3 Cells and OECs

To elucidate the endocytic mechanism and to investigate the effects of aromatic components combined on the endocytosis pathway of PLGA-NPs, various endocytosis inhibitors were used in this section. Briefly, bEnd.3 cells were pretreated for 2 h at 37 ± 0.5 °C with different types of endocytosis inhibitors separately (5 μg/mL Filipin III.5 μmol/L Cytochalasin D.6 μg/mL Chlorpromazine (CPZ)). The bEnd.3 cells treated with no inhibitors were used as controls. For the experimental group, the samples with endocytosis inhibitors were added to the medium, and bEnd.3 cells were cultured at 37 ± 0.5 °C for 6 h. At the same time, the final concentrations of the formulation solution were allowed to be consistent. Finally, bEnd.3 cells were harvested and analyzed by microplate reader. As for the endocytic mechanism in OECs, when OECs were sorted into 96 wells with 8 × 10^4^ cells, OECs were replaced with the medium containing endocytosis inhibitor when cultivated to 5 days. Endocytosis inhibitors were incubated with OECs for 1 h, following which formulations containing medium which contained endocytosis inhibitors was added and withdrawn after 2 h. Then, the next steps were the same as in the study of the uptake behavior of PLGA-NPs combined with aromatic components in OECs.

### 4.3. Statistical Analysis

All data were expressed as mean ± SD. Student’s *t*-test or ANOVA was employed for statistical analysis using IBM SPSS V27.0, with *p* < 0.05 indicating that the difference was statistically significant.

## Figures and Tables

**Table 1:** Effect of PLGA-NPs combined with menthol or curcumol on the average fluorescence intensity of four tight junction proteins in bEnd.3 cells monolayer.

Group	Average Fluorescence Intensity (%)			
	ZO-1	Occludin	Claudin-1	F-Actin
Control	185.4 ± 4.3	167.7 ± 1.2	164.7 ± 1.9	182.6 ± 5.4
PLGA-NPs	182.7 ± 4.2	162.9 ± 6.4	143.7 ± 3.8 **	141.1 ± 4.4 **
PLGA-NPs + Curcumol	143.5 ± 0.5 **^, ##^	129.5 ± 18.4 ^#^	113.5 ± 14.6 *^, #^	142.2 ± 2.0 **
PLGA-NPs + Menthol	135.9 ± 2.5 **^, ##^	132.9 ± 13.4 *^, #^	115.9 ± 10.9 **^, #^	90.1 ± 0.7 **^, ##^

Data were presented as mean ± SD (*n* = 3). * *p* < 0.05, ** *p* < 0.01 vs. Control; ^#^ *p* < 0.05, ^##^ *p* < 0.01 vs. PLGA-NPs.

**Table 2:** Effect of PLGA-NPs with aromatic components menthol or curcumol on the surface roughness of OECs and bEnd.3 cells.

Cell Type	Group	Rq (nm)	Ra (nm)	Young’s Modulus (MPa)
OECs	Control	23.5 ± 1.5	16.7 ± 1.3	15.9 ± 0.2
	PLGA-NPs	34.2 ± 1.9 **	26.7 ± 1.5 **	18.7 ± 0.6 **
	PLGA-NPs + Menthol	45.8 ± 2.6 ^##^	36.3 ± 1.5 ^##^	8.2 ± 0.7 ^##^
	PLGA-NPs + Curcumol	62.6 ± 2.6 ^##^	51.6 ± 2.1 ^##^	8.3 ± 1.0 ^##^
bEnd.3 cells	Control	20.7 ± 0.6	16.1 ± 0.7	17.6 ± 0.6
	PLGA-NPs	33.8 ± 3.3 **	26.5 ± 3.0 **	21.5 ± 0.6 **
	PLGA-NPs + Menthol	92.2 ± 2.5 ^##^	74.7 ± 1.9 ^##^	4.3 ± 1.3 ^##^
	PLGA-NPs + Curcumol	50.8 ± 8.6 ^##^	39.9 ± 6.7 ^##^	11.4 ± 1.0 ^##^

Data were mean ± SD (*n* = 5). ** *p* < 0.01 vs. Control; ^##^ *p* < 0.01 vs. PLGA-NPs.

## Data Availability

Data will be made available on request.

## Abbreviations

**Abbreviations**	**Full name in English**
AFM	Atomic force microscope
ALog P	Oil–water partition coefficient
BBB	Blood brain barrier
BCA	Bicinchonininc acid
bEnd.3 cells	Brain-derived endothelial cells.3
BPE	Bovine Pituitary Extract
CNS	Central nervous system
CPZ	Chlorpromazine
DAPI	4,6-diamidino-2-phenylindole
DMSO	Dimethyl sulfoxide
DMEM	Dulbeccos modified eagle medium
EMA	European Medicine Agency
FAK	Focal adhesion kinase
FBS	Fetal bovine serum
FDA	Food and Drug Administration
FITC	Fluorescein isothiocyanate
HBSS	Hanks’ Balanced Salt Solution
IN	Intranasa
MRPs	Multidrugresistance-associated proteins
MTT	3-(4,5-dimethylthiazol-2-yl)-2,5-diphenylte-trazolium bromide
NPs	Nanoparticles
OECs	Olfactory ensheathing cells
Papp	Apparent permeability coefficients
PBS	Phosphate buffer saline
PLGA	Poly (lactic-co-glycolic acid)
PLGA-NPs	Poly(lactic-co-glycolicacid)-Nanoparticles
PLGA-FITC	Poly(lactic-co-glycolicacid)-Fluorescein isothiocyanate
PDI	Polydispersity index
PVA	Polyvinyl alcohol
Ra	Roughness average
Rq	RMS roughness
TCM	Traditional Chinese Medicine
TEER	Transepithellal electric resistance
TEM	Transmission electron microscope
TJs	Tight junctions
US	The United States of America
WHO	World Health Organization
ZO-1	Zonula occludens 1
